# Supplementary material for: Regional Perspective of Antimicrobial Stewardship Programs in Latin American Pediatric Emergency Departments
Source: Antibiotics (Basel). 2023 May 16;12(5):916. doi: 10.3390/antibiotics12050916 (PMC10215519; doi:10.3390/antibiotics12050916)
Supplement: Supplementary file 1 [file antibiotics-12-00916-s001.zip › antibiotics-2276329-Supplementary File S1.pdf]

Supplemental Material. Survey of Antibiotic Stewardship in Pediatric Emergency Departments of Latin America

Antibiotic Stewardship in Pediatric Emergency Departments: A Survey about the Presence of this programs in Latin America

Background

Antibiotic stewardship programs are essential to reduce the infections of super-resistant microorganisms in hospital settings. In the pediatric population, antibiotic over-prescription is frequent without any evidence-based management, especially in the ambulatory setting. Information about these programs in Latin America is limited and the lack of data available in the pediatric emergency departments is scarce. The main objective of this survey was to get information about the presence of antibiotic stewardship programs in the pediatric emergency departments of Latin America and the use of clinical pathways for common infectious diseases in these settings.

Methods:

An electronic, anonymous, survey created and stored on Google Forms was sent to physicians (pediatricians and pediatric emergency physicians) in Latin America from the Pediatric Emergency Medicine Society (Sociedad Latino Americana de Emergencias Pediátricas -SLEPE) during the month of April 2023. The survey was in Spanish and included 6 questions about, the country, the type of hospital, the presence or not of antibiotic stewardship programs involving the emergency department, members in charge of the programs in their hospital, and the presence of protocols involving the use of antibiotics. The survey was sent to 52 physicians from 12 countries.

Results

The survey was sent to 52 physicians from different hospitals in LA and had a return rate of 31(59%) surveys from Argentina, Brazil, Bolivia, Chile, Colombia, Costa Rica, Dominican Republic, Guatemala, Mexico, Paraguay, Perú, and Uruguay. Most of the hospitals (85%) were tertiary care academic hospitals.

Of the total hospitals, 61.2% of them have activities related to Antibiotic Stewardship Program in their institution, but only 35% of them include the emergency department. Seventy-five percent of the physicians that answered the survey mentioned that in their departments they have protocols for different diseases that require antibiotics, like urinary tract infections, community-acquired pneumonia, and skin infections.

Regarding the members of the committee in charge of AS programs, all included an infectious disease physician and a smaller proportion of epidemiologists (52.6%), pediatricians (31.5%), microbiologists (26.3%), and pharmacists (26.3%). This varied from hospital to hospital. Hospitals, where no AS programs exist, were in Guatemala, Bolivia, México, and Chile. All the information regarding the experience of AS programs or activities related to pediatric Latin-American hospitals gathered from the survey is not published in peer review journals according to the physicians.

Table S1. Members of the Antibiotic Stewardship in Hospitals with pediatric emergency Departments in Latin America

Members of AS Programs	N=19<br>n (%)
**Pediatric Infectious Disease Physician**	19 (100)
**Epidemiologist**	10 (52.6)
**Pediatricians**	6 (31.5)
**Pharmacists**	5 (26.3)
**Microbiologist**	5 (26.3)
**Pediatric Intensivist Physician**	2(10.5)

Discussion

Antimicrobial stewardship programs are crucial in ensuring that antibiotics are used effectively and are becoming increasingly important in minimizing the threat of antibiotic resistance in the region. According to our survey 61.2% report AS activities in their hospital which is like what was reported by Muñoz et al. They reported in a survey done of physicians from 10 LA hospitals, 59% did have an AS program implemented but with limited resources. [1]

One common approach to AS programs in the emergency departments is the implementation of clinical pathways for specific conditions that include guidelines for antibiotic use. It is common the use clinical pathways in pediatrics and are developed to focus on specific infections like community-acquired pneumonia [2], bone infections [3], or skin and soft-tissue infections [4]. In our survey, 75% of the physicians mentioned the use of clinical protocols for common conditions seen in the emergency department, making this the main activity related to antimicrobial stewardship.

In regards to the members of the AS programs all of them mentioned that Infectious disease physicians were part of them, but there was a lot of variability in the rest of the members of the team. A recent statement from the Spanish Society of Paediatric Infectious Diseases mentioned that is crucial that a specialist in pediatric infectious

disease led the program but the team should include microbiologists, pharmacists, specialists in preventive medicine, nurses, and prescribers. [6]

There are several limitations of this survey since although a sample of Latin American countries was achieved, some countries have more than one Children's Hospital with different health systems and this might decrease the validity of the study. In addition, there was great variability in the information received.

Conclusion

Information about AS programs and activities in the pediatric emergency departments in Latin America is scarce and this limits the data available. Studies on antibiotic stewardship in these settings should focus on developing clinical pathways and incorporating physicians working in these departments into the hospital program initiatives.

References

1. Muñoz, J.S.; Motoa, G.; Escandón-Vargas, K.; Bavestrello, L.; Quiros, R.; Hernandez, C.; Pallares, C.; Villegas, M.V. Current Antimicrobial Stewardship Practices in Latin America: Where Are we? *Open Forum Infect. Dis.* **2015**, *2*, 192.
2. Tannous, R.; Haddad, R.N.; Torbey, P.H. Management of Community-Acquired Pneumonia in Pediatrics: Adherence to Clinical Guidelines. *Front. Pediatr.* **2020**, *8*, 302.
3. Woods, C.R.; Bradley, J.S.; Chatterjee, A.; Copley, L.A.; Robinson, J.; Kronman, M.P.; Arrieta, A.; Fowler, S.L.; Harrison, C.; Carrillo-Marquez, M.A.; et al. Clinical Practice Guideline by the Pediatric Infectious Diseases Society and the Infectious Diseases Society of America: 2021 Guideline on Diagnosis and Management of Acute Hematogenous Osteomyelitis in Pediatrics. *J. Pediatr. Infect. Dis. Soc.* **2021**, *10*, 801–844.
4. Tien, I. Update on the management of skin, soft-tissue, and osteoarticular infections in children. *Curr. Opin. Pediatr.* **2006**, *18*, 254–259.
5. Mistry, R.D.; May, L.S.; Pulia, M.S. Improving Antimicrobial Stewardship in Pediatric Emergency Care: A Pathway Forward. *Pediatrics* **2019**, *143*, e20182972.
6. Goycochea-Valdivia, W.A.; Pérez S.M.; Aguilera-Alonso, D.; Escosa-Garcia, L.; Campos L.M.; Baquero-Artigao, F.; Grupo de Trabajo PROA de la Sociedad Española de Infectología Pediátrica (SEIP). Position statement of the Spanish Society of Paediatric Infectious Diseases on the introduction, implementation and assessment of antimicrobial stewardship programmes in paediatric hospitals. *An. Pediatría* **2022**, *9*, 351.e1–351.e12.
